# Test–Retest Reliability of a Performance-Based Test Battery in Patients with Fibromyalgia According to Socio-Occupational Status

**DOI:** 10.3390/medsci14020236

**Published:** 2026-05-04

**Authors:** José Luis Socorro-Cumplido, Blanca Roman-Viñas, Miriam Almirall, Judith Sánchez-Raya, Josep Blanch-Rubió, Maria José Castro, Maria Giné-Garriga, Patricia Launois, Tamara Libertad Rodríguez Araya, Anna Arias Gassol, Raimon Milà Villarroel, Joaquim Chaler

**Affiliations:** 1 Blanquerna School of Psychology, Education and Sports Sciences, Universitat Ramon Llull, 08022 Barcelona, Spain; joseluissc1@blanquerna.url.edu (J.L.S.-C.); mariagg@blanquerna.url.edu (M.G.-G.); 2 EUSES University School of Health and Sports, University of Girona, 17190 Salt, Spain; 3 Rheumatology Department, University Hospital Vall d’Hebron, 08035 Barcelona, Spain; miriam.almirall@vallhebron.cat; 4 Physical Medicine and Rehabilitation, Hospital Vall d’Hebron, 08035 Barcelona, Spain; judith.sanchez@vallhebron.cat (J.S.-R.); patricia.launois@vallhebron.cat (P.L.); 5 Rheumatology Department, Hospital del Mar, 08003 Barcelona, Spain; jblanch@psmar.cat; 6 Clinical Expertise Unit for Central Sensitization Syndromes, Hospital del Mar, 08003 Barcelona, Spain; jcastro@psmar.cat; 7 Blanquerna School of Health Sciences, Universitat Ramon Llull, 08025 Barcelona, Spain; raimonmv@blanquerna.url.edu; 8 Rheumatology Department, Hospital Clinic, 08036 Barcelona, Spain; tlrodriguez@clinic.cat (T.L.R.A.); arias@clinic.cat (A.A.G.); 9 Physical Medicine and Rehabilitation Department, Hospital Egarsat, 08022 Barcelona, Spain; quim.chaler@gmail.com; 10 EUSES University School of Health and Sports, University of Girona, 08907 L’Hospitalet de Llobregat, Spain

**Keywords:** psychometric, objective physical performance, measurement stability, disability claim, patient outcome assessment

## Abstract

Background: Performance-based tests (PBTs) objectively assess functional capacity and are increasingly applied in fibromyalgia (FM) to complement patient-reported outcomes (PROMs). However, evidence regarding their reliability, especially considering patients’ socio-occupational status, is limited. This study aimed to determine test–retest reliability of a standardized PBT battery in women with FM and to examine the influence of employment status on measurement stability. Methods: A total of 119 women were assessed (89 with FM). The battery included the 6 min walk test (6MWT), handgrip strength test (HST), and 8 feet up and go test (8FUGT). Test–retest reliability was examined using the intraclass correlation coefficient (ICC), standard error of measurement (SEM), and smallest real difference (SRD). Analyses were conducted for the total FM group and socio-occupational subgroups (actively working, claiming disability, and permanent disability). Results: All PBTs demonstrated excellent test–retest reliability. Measurement stability was consistently higher in controls. Absolute reliability indices confirmed acceptable measurement stability. However, the claiming disability group showed markedly higher SEM% and SRD% for HST, suggesting reduced reproducibility. The 6MWT and 8FUGT maintained excellent reliability and stability across all groups. PROMs showed good-to-excellent reliability. Conclusions: PBTs showed excellent reliability in women with FM. However, reliability varied across socio-occupational groups, particularly for HST in patients claiming disability. PROMSs showed lower reliability than PBTs.

## 1. Introduction

Fibromyalgia (FM) is one of the most common musculoskeletal disorders, especially among women [1]. FM symptoms, characterized by chronic widespread pain, contribute to functional decline [2,3] and compromise participation, mainly in occupational activities [4,5,6]. A Spanish study estimated that the annual social cost associated with FM ranges from 2443.6 to 6183.8 million € [7]. A cohort study in Denmark showed that 50% of the FM patients were receiving disability pension 10 years after diagnosis [8]. In this context, an individualized patient-centered treatment requires comprehensive assessment.

The current diagnosis of FM follows the American College of Rheumatology (ACR) [9] criteria, which incorporate subjective measurements of the disease. However, as these approaches do not precisely define the clinical and functional situation of FM patients, a gold standard method for diagnosis remains absent [10]. Consequently, treatment strategies continue focusing largely on symptomatic relief, partly due to the limited understanding of the pathophysiology of the disease [11], as well as the real functional impact of symptoms [12].

Performance-based tests (PBTs), which evaluate physical capacity under standardized conditions, show a promising complementary approach to patient-reported outcome measurements (PROMs) and conventional clinical assessment. When incorporated into the clinical assessment, they can better characterize a patient’s clinical and functional status to guide an individualized treatment plan, monitor and evaluate treatment outcomes, and provide an objective, quantifiable basis for determining functional limitations. The latter is a challenging endeavor in FM patients due to important medicolegal implications. In this context, the International Classification of Functioning, Disability and Health (ICF) provides a comprehensive framework to conceptualize and assess functioning, supporting the selection and interpretation of PBTs across relevant domains [13].

Yet, despite PBT’s potential and widespread use in FM, there is no consensus on which tests are more accurate to measure changes over time [14,15], and rigorous validation studies targeting the FM population are limited. Key psychometric aspects such as reliability, validity, responsiveness, and potential measurement bias are often overlooked or insufficiently reported [16].

Given that PBTs and PROMs capture different but complementary aspects of functioning, understanding their respective reliability and measurement behavior is essential to optimize their combined use in clinical and research settings. Despite these advances, important gaps remain.

In addition, to the best of our knowledge, no studies have explored the influence of disability-related status on the psychometric properties of those tests. For instance, disability claiming may introduce additional unique psychosocial and behavioral variables that could impact test performance, interpretation, and ultimately its utility [14,15]. Thus, there is an urgent need for rigorous methodological studies to determine the reliability and stability of PBT measurements in FM patients, including subgroups claiming disability. They would allow clinicians to accurately plan treatments, monitor the disease, and evaluate the functional impact of the disease and applied therapies [16].

Consequently, the first objective is to assess the psychometric characteristics related to the reliability of some PBTs in FM patients. A secondary objective is to assess the impact of the socio-occupational status (patients actively employed, on sick leave, and/or claiming disability and with recognized disability) on reliability parameters and, finally, to compare the psychometric parameters of PBTs and PROMs.

## 2. Materials and Methods

### 2.1. Study Design

An observational test–retest study was designed to assess the reliability of three PBTs in patients with FM. The tests selected were the 6 min walk test (6MWT), handgrip strength test, and the 8 feet up and go test. These PBTs were selected because they cover the main functional domains most frequently impaired in FM—aerobic capacity (6MWT), muscular strength (handgrip strength test, 8 feet up and go test), and dynamic balance/mobility (8 feet up and go test)—and have shown feasibility, safety, and good psychometric properties in chronic pain populations, including FM [17,18,19]. Moreover, these PBTs are widely recommended in both rehabilitation and occupational settings for their clinical interpretability, minimal equipment requirements, and capacity to detect functional limitations relevant to daily life activities [20,21]. Test–retest reliability was evaluated following the Consensus-based Standards for the selection of health Measurement Instruments (COSMIN) guidelines [22].

### 2.2. Participants

Patients with FM were recruited from 4 hospitals in Barcelona (Hospital Clínic, Hospital Egarsat, Hospital del Mar, and Hospital de la Vall d’Hebron) between February 2024 and April 2025 by e-mail or telephone. Additionally, a control group of women without FM was included to provide a reference framework for interpreting reliability, allowing comparison with a population not affected by the condition. Control participants were recruited in the city of Barcelona through informational leaflets distributed in hospitals, primary health care settings, and universities. Those who agreed to participate contacted the research team via email and were screened for eligibility before inclusion.

This study was conducted in accordance with the ethical principles of the Declaration of Helsinki and complied with the applicable national regulations on biomedical research. Ethical approval was obtained from all participating institutions. The protocol was reviewed and approved by the Clinical Research Ethics Committee of Parc de Salut Mar (CEIm-PSMAR; reference 2024/11477/I; date of approval: 25 July 2024), the Clinical Research Ethics Committee of Hospital Clínic de Barcelona (Reg. HCB/2024/1032; date of approval: 8 October 2024), the Ethics Committee of the Fundació Assistencial Mútua Terrassa (reference P/21-082; date of approval: 9 February 2024), and the Ethics and Research Committee of the Ramon Llull University (reference 2223019D; date of approval: 16 June 2024). Each committee evaluated and approved the protocol, the patient information sheet, and the informed consent form. Written informed consent was obtained from all participants prior to inclusion. The study was prospectively registered in ClinicalTrials.gov under the identifier NCT06819930.

A total of 87 patients and 30 control subjects agreed to participate in the study.

The participants fulfilled the following inclusion criteria: (a) female between 30 and 65 years old, (b) diagnosed with FM by a rheumatologist according to the ACR criteria [9], (c) understand the PBT protocols, and (d) be able to communicate effectively with the study researchers. Exclusion criteria were the following: (a) to show illiteracy and/or lack of understanding of Spanish, (b) to have severe psychiatric and/or psychological disorders, (c) to have other rheumatological, autoimmune, neurological, or musculoskeletal conditions that could affect physical performance (e.g., inflammatory arthritis, systemic autoimmune diseases, or neurological disorders affecting mobility), and (d) patients who refused to participate in the study.

### 2.3. Procedure

On day one, anthropometric measurements were recorded. Height (m) was measured using a stadiometer (Seca 22, Hamburg, Germany) and weight (kg) with a scale (InBody 720, Biospace, Seoul, Republic of Korea). Body mass index (BMI) was calculated as weight (kg) divided by height squared (m^2^). Socio-demographic data were collected, including age, ethnicity, marital status, educational level, and socio-occupational status (actively employed, paid sick leave, claiming disability, and/or recognized disability). Furthermore, the Widespread Pain Index (WPI) and Symptom Severity Scale (SSS) [9], the Spanish version of the Revised Fibromyalgia Impact Questionnaire (FIQR) [23], the 36-item Short-Form Health Survey (SF-36) [24], and the Hospital Anxiety and Depression Scale (HADS) [25] were administered. Following the completion, participants performed the three PBTs in a standardized order. The sequence (handgrip strength test, 8 feet up and go test, and 6MWT) was selected to minimize fatigue and carry-over effects, progressing from lower to higher physical demand to avoid interference between tests [18]:

1. The handgrip strength test was used to assess muscle power functions (code b730) described in ICF [26,27]. The test was measured using a handheld calibrated dynamometer (Jamar^®^ dynamometer, Saehan Corporation, Masan, Republic of Korea). Each participant performed the test sitting on a chair, shoulder at 0° of adduction (arm against the trunk), elbow flexed to 90°, and forearm and wrist in neutral position, without support. Once positioned, participants were asked to squeeze the dynamometer and perform a maximum sustained contraction for 3 s as strongly as they could. Three measurements were performed with both hands with 60 s rest periods between them to avoid fatigue [28,29]. The mean value of the three trials for each hand was noted (Kg).
2. The 8 feet up and go test is a well-established measure of overall functional mobility, encompassing changing basic body positions as described in the ICF (code d410) [26,27] as well as coordination and balance. Involves standing up from a chair, walking 2.44 m to and around a cone, and returning to the chair in the shortest possible time without running [20]. The test was performed three times, with 60 s rest periods between trials to reduce the potential impact of fatigue or pain on performance consistency. The mean time of three trials was recorded and used in the analyses (seconds).
3. The 6MWT was used to measure functional exercise capacity through cardiorespiratory fitness, corresponding to exercise tolerance functions (code b455) and walking (code d450) within the ICF framework [26,27]. Participants were instructed to walk safely and comfortably as quickly as possible along a 45.7 m rectangular course for six minutes. The maximum distance (in meters) walked was registered. This test has been shown to have good reliability in women with FM [18,30].

Prior to the formal reliability assessment, a familiarization session was conducted immediately prior to the first testing (day 1), to ensure all participants were adequately acquainted with the PBTs and to minimize potential learning effects that could influence performance across testing sessions. Given that individuals with FM often experience pain, fatigue, and cognitive difficulties, prior exposure to the test procedures aimed to enhance the reliability of the measurements by reducing variability associated with unfamiliarity or apprehension. Each test was conducted following standardized protocols, with verbal encouragement provided according to predefined criteria to maintain consistency. During familiarization, participants performed one submaximal trial of each test to ensure understanding of the procedures. Sufficient rest intervals were allowed between tests to prevent excessive fatigue from influencing subsequent performances.

On testing day 2, scheduled approximately 10 days after testing day 1, participants completed the same questionnaires. Prior to this assessment, participants were contacted to confirm clinical stability. In case of temporary symptom exacerbation, the assessment was rescheduled to avoid the influence of acute fluctuations. As a result, minor variations in the test–retest interval were allowed to ensure stable clinical conditions. Additionally, to assess the intra-rater and test–retest reliability, the three PBTs were repeated in the same standardized order. All assessments were performed by the same trained evaluator to ensure consistency in test administration and to reduce potential bias and ensure the validity of the intra-rater and test–retest reliability assessments. The rater was blinded to the scores obtained in previous assessments by ensuring that data from prior measurements were not accessible at the time of reassessment. Each evaluation was conducted independently, with a sufficient time interval between sessions to minimize recall bias. These precautions aimed to prevent any influence of previous scores on subsequent measurements, thereby enhancing methodological rigor.

Standardized protocols were followed in both sessions, maintaining the same environmental conditions, time of the day, instructions, and order of testing to minimize external variability. Participants were instructed to perform the tests at their maximum capacity while ensuring safety precautions, and to maintain their usual physical activity level and refrain from excessive exertion 24 h prior to each assessment. Additionally, participants were required to be in a clinically stable condition, without acute exacerbations between assessments, and were instructed to maintain their usual medication and daily habits throughout the study period.

### 2.4. Statistical Analysis

Based on normality tests (Shapiro–Wilk and Kolmogorov), parametric tests were employed. Descriptive statistics (mean and standard deviation [SD]) of age and anthropometric measurements were calculated for the whole sample and according to socio-occupational status.

To estimate the intraclass correlation coefficient (ICC) and its 95% confidence intervals of the 6MWT, handgrip strength test and 8 feet up and go test at test and retest times, the 3.1 (two-way mixed effects, consistency, single rater/measurement) model was used [31,32]. This model was selected because all measurements were performed by the same evaluator under standardized conditions, and the primary objective was to assess the consistency and stability of individual rankings across repeated assessments rather than absolute agreement. Regarding the ICC classification, an ICC value lower than 0.50 indicates “poor” reliability, an ICC value between 0.50 and 0.75 indicates “moderate” reliability, an ICC value between 0.75 and 0.90 indicates “good” reliability, and an ICC value higher than 0.90 indicates “excellent” reliability [32].

Absolute reliability was determined by computing the standard error of measurement (SEM), which is calculated as SEM = SD × √1-ICC, where SEM is the SEM and SD is the mean SD of the two outcomes (test and retest). The smallest real difference (SRD) was calculated as SRD = 1.96 × SEM × √2. To facilitate the comparability of errors of measurement, we have calculated the SEM and SRD indices as percentages as follows: SEM% = (SEM/mean of test and retest) × 100 and SRD% = (SRD/mean of test and retest) × 100. As no universal benchmarks for patients with FM exist for relative measurement error, previous studies in musculoskeletal and neurological populations have suggested that SEM% values below 10% and SRD% values below 30% may be considered acceptable [33,34].

To identify the level of agreement between the test and retest, and the measuring devices in the 6MWT, handgrip strength test and 8 feet up and go test, Bland–Altman plots were performed [35].

The statistical significance was established at the *p* ≤ 0.05 level. Statistical analysis was conducted using the Statistical Package for the Social Sciences (SPSS, version 24.0; IBM Corp., Armonk, NY, USA) and R software (version 4.4.1) as needed.

Based on socio-occupational status, patients were categorized into (1) patients with FM who were actively working (n = 30); (2) patients with FM on sick leave and claiming disability or under evaluation to attain permanent disability compensation (n = 30); (3) patients with FM with permanent work disability (officially recognized by the Spanish National Institute of Social Security, INSS) (n = 27); and (4) a control group of sedentary women without FM (n = 30).

## 3. Results

**Figure 1:**
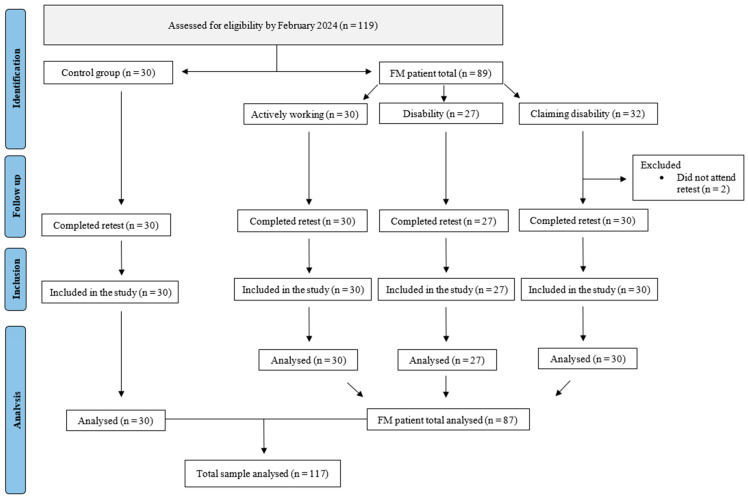
Flowchart of participants’ recruitment, inclusion, and analysis. Abbreviations: FM, fibromyalgia; n, number of participants.

A total of 119 participants were initially assessed for eligibility. Two FM participants (claiming disability group) did not attend day 2 and were excluded, resulting in a final sample of 87 FM patients and 30 controls (Figure 1). Demographic and clinical baseline characteristics of all participants are summarized in Table 1, and FM subgroups in Table 2.

Note that the only significant differences between FM patients and controls were found in weight (*p* < 0.001), BMI (*p* < 0.001), and marital status (*p* = 0.027) (Table 1). Additionally, educational level differed both between controls and FM patients (*p* < 0.001) (Table 1) and among FM subgroups (*p* = 0.005) (Table 2).

### 3.1. Reliability of PBTs: Control and FM Total Groups

All PBTs demonstrated excellent test–retest reliability across groups, with ICC values exceeding 0.90. However, absolute reliability metrics and Limits of Agreement (LoA), although within the acceptable level, revealed substantial group-dependent differences (Table 3).

#### 3.1.1. Six Minute Walk Test

Both the control group and FM group showed ICC values in the range of excellent reliability level (>0.90), although the latter showed slightly lower values (Table 3). Regarding SEM, SRD, and LoA, the FM group values almost doubled those found in the control group, thus indicating a relatively lower measurement stability. However, SEM% and SRD% were within acceptable ranges in both groups (<10% and <30%, respectively) (Table 3).

#### 3.1.2. Handgrip Strength Test

Handgrip strength test, both in dominant and non-dominant hands, yielded excellent ICCs in both control and FM groups (Table 3). Nevertheless, FM patients showed substantially larger SEM and SRD, close to the cut-point, indicating unacceptable levels in both hands (>10% for SEM% and >30% for SRD%) (Table 3). It is noteworthy that the dominant hand SEM% and SRD% values in FM patients were almost three times higher than those found in healthy volunteers.

#### 3.1.3. Eight Feet Up and Go Test

The 8 feet up and go test demonstrated excellent reproducibility in both control and FM groups, with ICC values exceeding 0.90 (Table 3). Regarding absolute reliability indices, controls exhibited more favorable results than FM patients; however, both SEM% and SRD% remained within acceptable thresholds in each group, indicating adequate measurement stability.

### 3.2. Reliability of PBTs Battery by Socio-Occupational Status Within the FM Cohort

Subgroup analyses showed excellent test–retest reliability for all the PBTs across all the FM subgroups, with ICC values exceeding 0.91 in every case. However, absolute reliability indices (SEM, SEM%, SRD, and SRD%) and Limits of Agreement (LoA) varied across the three groups, highlighting relevant differences in measurement accuracy and within-subject variability in some cases.

#### 3.2.1. Six Minute Walk Test

Test–retest reliability was excellent across subgroups, with ICCs ranging from 0.914 (disability group) to 0.985 (claiming disability group) (Table 4).

As for the absolute reliability parameters, they were within the levels of acceptance in all cases. The group with the lowest SEM and SRD was the one claiming disability (Table 4).

#### 3.2.2. Handgrip Strength Test

ICCs ranged from 0.922 to 0.978 across subgroups and dominant/non-dominant hands. The disability group showed the highest ICCs for both dominant (0.978) and non-dominant hand (0.974), suggesting highly reliable measurements in this group. The disability group showed the lowest SEM and SRD values, followed by the actively working group values; SEM% and SRD% were in all cases within the acceptable range (Table 4). On the other hand, the group claiming disability showed the highest SEM and SRD records, which rendered both SEM% and SRD% values in both hands well above the commonly accepted limits (Table 4), thus indicating a poor stability of the measurement.

#### 3.2.3. 8 Feet Up and Go Test

Test–retest reliability was excellent across subgroups, with ICCs ranging from 0.948 (disability group) to 0.991 (claiming disability group). As for the SEM and SRD, the group of patients claiming disability showed the lowest values. However, in all groups, the SEM% and the SRD% were within the acceptable limits (Table 4).

### 3.3. Reliability of Patient Reported Outcomes—Whole Group Analysis

FIQR TS, HADS total score, and its subscales (anxiety and depression) showed excellent ICC values (namely, above 0.9 in all cases). In contrast, the FIQR subscale’s function, overall impact, and symptoms, as well as WPI, SSS, and both SF-36 physical and mental components, show ICCs within the “good” range of values (Table 5). Most SEM and SRD values registered were within the acceptable range. The only exception was for the overall impact subscale of FIQ-R, which attained SEM% and SRD% values of 13.86 and 38.41, respectively.

### 3.4. Reliability of Patient Reported Outcomes—Subgroup Analysis

#### 3.4.1. Revised Fibromyalgia Impact Questionnaire

Both the disability and actively working groups showed excellent ICC values, whereas patients claiming disability reached a good level. In all cases, however, SEM, SRD, and their % were within acceptable ranges (Table 6). As for the subscales, the disability group showed the best ICC values relative to function and overall impact (excellent and good, respectively). On the other hand, ICCs registered in the other groups were within the good and moderate level of reliability for function and overall impact, respectively, in both groups. Symptom subscale ICCs indicated a good reliability in the three groups (Table 6). As for absolute parameters, the SEM and SRD lower values for all subscales were also found in the disability group, whilst the higher ones were found in active patients. The disability group SEM% and SRD% values were within the limits of acceptance in all cases. Additionally, function and symptoms subscales in active and claiming disability groups showed SEM% and SRD% values within the acceptance limits. Finally, the overall impact subscale of the latter groups’ SEM% and SRD% values were well above the acceptance limits, especially in the active patient group (Table 6). In brief, the finding indicates a poor stability of the overall impact subscales measurement in both subgroups (active and claiming disability).

#### 3.4.2. Widespread Pain Index

All subgroups presented ICCs within the good reliability range. The higher value was observed in the disability group. Absolute parameters were within acceptable ranges for the three groups, whereas the active patients showed values slightly above the acceptance level for SEM% (Table 6).

#### 3.4.3. Symptom Severity Scale

The actively working patient group showed the higher ICC value (within the good reliability range), whereas those claiming disability or with recognized disability showed values in the moderate reliability level (Table 6). On the other hand, the SEM% and SRD% were within the acceptable range in all groups except for the active patient SEM%, which was slightly above the acceptance level (Table 6).

#### 3.4.4. The 36-Item Short-Form Health Survey

SF-36 physical component ICCs were within the good reliability range in all three groups; the highest value was observed in patients with disability. As for the SF-36 mental component, the disability and claiming disability groups showed the highest ICC values within the excellent reliability range. The active group ICC was at a good reliability level. Regarding the SEM% and SRD%, in all cases, the values were within the acceptable ranges (Table 6).

#### 3.4.5. Hospital Anxiety and Depression Scale

Both the disability and claiming disability groups showed ICCs in the range of excellent reliability, both for anxiety and depression aspects. The actively working patients’ ICC was in the range of good reliability for both aspects of the scale (Table 6). Regarding absolute parameters, the patients with disability or claiming disability showed SEM% and SRD% within the acceptable range. On the other hand, the active patients showed SEM% above the acceptable limits in both anxiety and depression subscales, and SRD% value above limits in the depression subscale, thus indicating an important instability of the depression measure through the HADS subscale in this group.

## 4. Discussion

All selected PBTs exhibited excellent relative reliability (ICC > 0.90) and acceptable measurement error indices in the overall FM cohort, with some important subgroups showing variability and lower stability than controls. The inclusion of a control group provides a reference framework to distinguish disease-related variability from normal measurement variability, which is essential for interpreting reliability outcomes of PBTs in FM [36,37]. As for the absolute reliability parameters, the SEM% and SRD% of the 6MWT and the 8 feet up and go test showed a good level of acceptance in all groups, indicating an adequate stability of the measurement. The conspicuous exception was found in the handgrip strength test SEM% and SRD% values obtained in the claiming disability group, which widely exceeded the acceptance thresholds, indicating a poor stability of the measurement. Regarding the actively working and disability groups, the handgrip strength test SEM% and SRD% retained acceptable values.

Test–retest reliability of the 6MWT was excellent across all groups (ICC 0.91 to 0.98) and in line with previous studies that found ICC values from 0.733 to 0.98 [18,30,38]. Regarding absolute reliability parameter values, other studies found higher results, probably due to methodological issues, reporting SEM values that ranged from 17.1 to 33.5 m before an intervention, and decreased to 8.1 to 15.8 m after the intervention [39]. Also, previous research has reported a SEM value of 23.52 m and an SRD of 65.20 m [18]. To estimate the relevance of measurement instability, we calculated the SEM% and SRD% and found that controls showed very low parameter values, whereas FM groups showed a lower stability of measurement but still within acceptable limits. We did not find SEM% and SRD% calculations in other studies evaluating the psychometric properties of the 6MWT to compare.

For the handgrip strength test, the ICCs results exceeded 0.90, which is in accordance with previous investigations reporting ICCs ranging from 0.91 to 0.96 [18]. The same study included values of SEM (1.46 kg) and SRD (4.04 kg), but SEM% and SRD% were not calculated, and thus, acceptability is unknown. In our cohort, controls showed good reproducibility, whereas FM patients exhibited much higher error. The subgroup claiming disability showed the poorest absolute reliability, as opposed to the more acceptable values in those with permanent disability. This reduced absolute reliability may reflect the combined effect of pain, fatigue, and psychosocial factors that influence performance consistency in claiming disability patients with chronic pain conditions [40,41]. Beyond its psychometric implications, impaired and unstable handgrip performance in this subgroup has relevant clinical assessment implications. In fact, handgrip strength is recognized by the European Working Group on Sarcopenia in Older People 2 (EWGSOP2) consensus as the primary criterion for diagnosing probable sarcopenia [21]. Previous evidence has shown that individuals with FM have an increased risk of secondary sarcopenia and dynapenia, even in the absence of reduced muscle mass [42]. Moreover, chronic musculoskeletal pain has been associated with both diminished muscle strength and higher risk of sarcopenia, with potential repercussions for disability and cognitive decline [39]. Thus, the handgrip strength test, despite being essential as a diagnostic and prognostic tool, may be less stable in FM patient subgroups (i.e., claiming disability), reinforcing the need for registering the socio-occupational status, performing as many repeated measures as necessary, and being cautious with result interpretation in clinical settings and especially in disability-related situations.

The 8 feet up and go test also demonstrated excellent ICCs in our study (>0.90), in line with other studies (0.91 to 0.96) [18]. Again, percentages were not expressed, and only SEM (0.58 s) and SRD (1.60 s) were reported. To our knowledge, this is the first study to report SEM% and SRD% for the 8 feet up and go test. While reproducibility was very high in controls, FM patients showed higher error. Interestingly, the claiming disability subgroup achieved the best indices, whereas the actively working group showed poorer reproducibility.

More recent contributions in FM management [43,44] have reinforced the importance of standardized robust outcomes to capture clinically meaningful changes. The key domains consistently prioritized include pain, fatigue, sleep quality, and daily functioning, which are regarded as core outcomes in both clinical trials and routine care [45]. In this sense, our study not only corroborates but also strengthens the available evidence base by integrating rigorous COSMIN-guided reliability analyses, thereby providing a methodological model for future research and clinical application of PBTs in FM.

Unlike PBTs ICC (all excellent), most PROMs ICC values were within the immediately inferior range, a good level of reliability. In this case, the best reliability scores were registered in patients with disabilities, whilst the worst reliability values were registered in those actively working. This finding highlights the importance of systematically adding PBTs to PROMs to better understand the functional impact of FM [46]. Not surprisingly, clinical outcome assessments based on PBTs (which eliminate the judgement component of PROMs or clinician- or observer-reported measures) are increasingly advocated in trials design [47,48], particularly given the subjective nature and variability of symptom reporting in FM [11].

Our findings on the reliability of PROMs are aligned with a recent systematic review, which concluded that most FM-specific questionnaires, such as the FIQ-R, HADS, and SF36, generally demonstrate good to excellent psychometric properties, particularly in terms of internal consistency and test–retest reliability [49]. However, that review also highlighted an important limitation: although all of them measured unstable constructs, none of the studies reported error indices such as the SEM or SRD, and none provided percentage-based metrics (SEM% and SRD%) (namely, stability of measurement determination), thereby limiting interpretability and comparability across populations. In contrast, our study systematically calculated all four parameters (SEM, SRD, SEM%, and SRD%), thus providing a more comprehensive picture of both relative and absolute reliability. While ICCs confirmed good to excellent test–retest consistency for most PROMs, some scales—most notably the FIQR overall impact subscale—showed SRD% values well above acceptable thresholds, underscoring the importance of including absolute reliability indices.

Importantly, while previous studies have typically evaluated PROMs without stratification, our subgroup analyses revealed that socio-occupational status exerted a notable influence on measurement stability, with actively working patients consistently showing poorer reproducibility compared to those with recognized disability and claiming disability. To the best of our knowledge, this is the first study to examine the moderating effect of occupational status on the psychometric performance of PROMs in FM. This novel approach underlies the potential impact of socio-occupational factors on PROMs and highlights the need to consider these dimensions in both research and clinical practice when selecting and interpreting PROMs for this population.

From a clinical and practical perspective, our findings have important implications. The observed variability in measurement stability across socio-occupational groups, particularly in patients claiming disability, suggests that PBT results should be interpreted with caution in these contexts. In clinical practice, this highlights the need to complement single assessments with repeated measurements and to consider psychosocial and contextual factors when evaluating functional capacity. Moreover, in medicolegal settings, where objective measures are often used to support disability-related decisions, understanding the potential instability of certain tests, such as the handgrip strength test, becomes especially relevant to avoid misinterpretation of patient performance. These findings support a more nuanced and context-aware use of performance-based assessments in FM.

Furthermore, future research should aim to establish population-specific reference values for PBTs in FM, which would enhance the clinical interpretability of these measures.

This study has some limitations that should be considered. First, the sample included only women with FM recruited from specialized hospital settings, which may limit the external validity of the findings to male patients or community-based populations. Second, while intra-rater consistency was ensured by using the same evaluator in both assessments, inter-rater reliability was not evaluated and should be addressed in future research. Third, the benchmarks used to interpret the relative measurement error (SEM% < 10% and SRD% < 30%) were derived from studies conducted in diverse clinical pathologies and should, therefore, be regarded as indicative rather than definitive thresholds for FM. Finally, the study was conducted within a specific sociocultural and healthcare context, which may have influenced participants’ perceptions, behaviors, and access to healthcare resources. Therefore, caution should be exercised when generalizing these findings to populations from different geopolitical or sociocultural settings, where contextual factors could lead to variations in test performance and reliability outcomes.

## 5. Conclusions

PBTs analyzed in the present study show an excellent level of reliability in assessing patients with FM functioning. However, specific socio-occupational status (i.e., persons claiming disability) may be associated with reduced reliability of the handgrip strength test in patients with FM, thus underlining the importance of a careful interpretation in the clinical setting. Finally, the lower reliability observed for PROMs compared to PBTs highlights the need for combining both assessment approaches in functioning assessment of patients with FM.

## Figures and Tables

**Table 1:** Participants’ baseline characteristics.

	Control Group (n = 30)	FM Total Sample (n = 87)	*p*-Value
	Mean (SD)	Mean (SD)	
Age (years)	49.57 (8.46)	52.62 (8.11)	0.081 ^1^
Height (m)	1.61 (0.07)	1.60 (0.06)	0.826 ^2^
Weight (kg)	61.32 (8.14)	72.05 (14.00)	<0.001 *** ^1^
BMI (kg/m^2^)	23.77 (2.98)	28.04 (5.35)	<0.001 *** ^1^
	n (%)	n (%)	
BMI classification (kg/m^2^)			
Underweight (<18.5)	0 (0.00)	2 (2.30)	0.002 ** ^3^
Normal weight (18.5–24.9)	21 (70.00)	27 (31.03)	
Overweight (≥25.0)	7 (23.33)	33 (37.93)	
Obesity (≥30.0)	2 (6.67)	25 (28.74)	
Ethnicity			
European	30 (100.00)	82 (94.25)	0.632 ^3^
Latin American	0 (0.00)	2 (2.30)	
Sub-Saharan	0 (0.00)	1 (1.15)	
Maghrebi	0 (0.00)	2 (2.30)	
Marital status			
Married or partnered	21 (70.00)	43 (49.43)	0.027 * ^3^
Not married or partnered	9 (30.00)	44 (50.57)	
Educational level			
Primary education	0 (0.00)	15 (17.24)	<0.001 *** ^3^
Secondary education	9 (30.00)	52 (59.77)	
Tertiary/University degree	21 (70.00)	20 (22.99)	

Note: *p*-values correspond to the following statistical tests: ^1^ Independent samples *t*-test, Student, ^2^ Mann–Whitney U test, ^3^ Pearson’s χ^2^ test. Abbreviations: BMI, Body mass index; FM, fibromyalgia; kg, kilograms; m, meters; SD, standard deviation. * *p* < 0.05, ** *p* < 0.01, *** *p* < 0.001, statistically significant differences.

**Table 2:** FM patient subgroups characteristics.

	Actively Working (n = 30)	Claiming Disability (n = 30)	Disability (n = 27)	*p*-Value
	Mean (SD)	Mean (SD)	Mean (SD)	
Age (years)	51.03 (9.87)	51.63 (7.04)	55.48 (6.39)	0.105 ^1^
Height (m)	1.61 (0.05)	1.62 (0.06)	1.59 (0.06)	0.138 ^2^
Weight (kg)	69.34 (12.44)	75.43 (15.48)	71.3 (13.65)	0.277 ^1^
BMI (kg/m^2^)	26.91 (5.03)	28.81 (5.17)	28.46 (5.84)	0.357 ^1^
	n (%)	n (%)	n (%)	
BMI classification (kg/m^2^)				
Underweight (<18.5)	0 (0.00)	0 (0.00)	2 (7.41)	0.243 ^3^
Normal weight (18.5–24.9)	13 (43.33)	8 (26.67)	6 (22.22)	
Overweight (≥25.0)	9 (30.00)	12 (40.00)	12 (44.44)	
Obesity (≥30.0)	8 (26.67)	10 (33.33)	7 (25.93)	
Ethnicity				
European	28 (93.33)	27 (90.00)	27 (100.00)	0.140 ^3^
Latin American	2 (6.67)	0 (0.00)	0 (0.00)	
Sub-Saharan	0 (0.00)	1 (3.33)	0 (0.00)	
Maghrebi	0 (0.00)	2 (6.67)	0 (0.00)	
Marital status				
Married or partnered	11 (36.67)	15 (50.00)	17 (62.96)	0.140 ^3^
Not married or partnered	19 (63.33)	15 (50.00)	10 (37.04)	
Educational level				
Primary education	1 (3.33)	6 (20.00)	8 (29.63)	0.005 ** ^3^
Secondary education	16 (53.33)	19 (63.33)	17 (62.96)	
Tertiary/University degree	13 (43.33)	5 (16.67)	2 (7.41)	

Note: *p*-values correspond to the following statistical tests: ^1^ Independent samples *t*-test Student, ^2^ Mann–Whitney U test, ^3^ Pearson’s χ^2^ test. Abbreviations: BMI, Body mass index; FM, fibromyalgia; kg, kilograms; m, meters; SD, standard deviation. ** *p* < 0.01, statistically significant differences.

**Table 3:** Reliability of PBTs in control group and FM total sample.

	Test	Retest	ICC (95%CI)	SEM	SEM%	SRD	SRD%	LoA
	Mean (SD)	Mean (SD)						
Control group (n = 30)								
6MWT (m)	526.13 (60.40)	525.43 (59.21)	0.977 (0.951–0.989)	9.07	1.73	25.14	4.78	−34.73; 36.13
HD (kg)	28.29 (5.22)	28.27 (5.67)	0.965 (0.927–0.984)	1.02	3.60	2.82	9.98	−3.89; 3.93
HND (kg)	25.82 (4.90)	25.52 (5.63)	0.956 (0.907–0.979)	1.10	4.30	3.06	11.93	−3.95; 4.56
8FUGT (s)	5.28 (0.69)	5.32 (0.71)	0.979 (0.955–0.990)	0.10	1.91	0.28	5.31	−0.43; 0.36
FM total (n = 87)								
6MWT (m)	346.97 (84.73)	346.54 (82.86)	0.962 (0.941–0.975)	16.33	4.71	45.28	13.06	−62.77; 63.62
HD (kg)	16.16 (7.26)	15.64 (7.20)	0.952 (0.926–0.968)	1.58	9.96	4.39	27.61	−5.57; 6.61
HND (kg)	14.83 (7.16)	14.46 (6.88)	0.959 (0.937–0.973)	1.42	9.71	3.94	26.90	−5.12; 5.86
8FUGT (s)	9.12 (3.10)	9.36 (3.12)	0.978 (0.967–0.986)	0.46	4.99	1.28	13.84	−2.02; 1.55

Note: ICC value and reliability level < 0.50, poor (red); 0.50–0.75, moderate (orange); 0.75–0.90, good (yellow); >0.90 excellent (green); %SEM value < 10%, acceptable (light green); >10%, poor (light yellow); %SRD value < 30%, acceptable (light green); >30%, poor (light yellow). Abbreviations: CI, confidence interval; FM, fibromyalgia; HD, Handgrip Test Dominant Side; HND, Handgrip Test Non-Dominant Side; ICC, intraclass correlation; LoA, Limits of Agreement; SD, standard deviation; SEM, standard error of measurement; SRD, smallest real difference; 6MWT, 6 min walking test; 8 FUGT, 8 feet up and go test.

**Table 4:** Reliability of PBTs in FM patient subgroups.

	Test	Retest	ICC (95%CI)	SEM	SEM%	SRD	SRD%	LoA
	Mean (SD)	Mean (SD)						
Actively working (n = 30)								
6MWT (m)	376.93 (69.67)	375.33 (83.61)	0.951 (0.897–0.977)	16.96	4.51	47.02	12.50	−63.46; 66.66
HD (kg)	19.53 (6.32)	18.81 (6.94)	0.933 (0.860–0.968)	1.72	8.95	4.76	24.81	−5.79; 7.23
HND (kg)	18.24 (6.37)	18.17 (6.24)	0.942 (0.879–0.972)	1.52	8.34	4.21	23.12	−5.70; 5.85
8FUGT (s)	7.95 (2.43)	8.23 (2.48)	0.948 (0.890–0.975)	0.56	6.92	1.55	19.18	−2.43; 1.86
Claiming disability (n = 30)								
6MWT (m)	335.03 (98.37)	336.17 (90.22)	0.985 (0.969–0.993)	11.55	3.44	32.01	9.54	−45.84; 43.57
HD (kg)	13.25 (7.13)	12.85 (6.65)	0.922 (0.836–0.963)	1.92	14.75	5.33	40.87	−6.86; 7.67
HND (kg)	12.14 (7.09)	11.50 (6.34)	0.939 (0.872–0.971)	1.66	14.03	4.60	38.89	−5.68; 6.94
8FUGT (s)	10.24 (4.16)	10.33 (4.04)	0.991 (0.982–0.996)	0.39	3.78	1.08	10.48	−1.57; 1.39
Disability (n = 27)								
6MWT (m)	326.93 (76.94)	326.07 (65.71)	0.914 (0.811–0.961)	20.92	6.41	57.98	17.76	−78.15; 79.85
HD (kg)	15.65 (7.06)	15.22 (6.92)	0.978 (0.951–0.990)	1.04	6.72	2.87	18.62	−3.61; 4.47
HND (kg)	14.02 (6.76)	13.61 (6.45)	0.974 (0.942–0.988)	1.07	7.71	2.95	21.37	−3.74; 4.55
8FUGT (s)	9.18 (1.70)	9.53 (2.13)	0.948 (0.886–0.976)	0.44	4.67	1.21	12.94	−2.03; 1.33

Note: ICC value and reliability level < 0.50, poor (red); 0.50–0.75, moderate (orange); 0.75–0.90, good (yellow); >0.90 excellent (green); %SEM value < 10%, acceptable (light green); >10%, poor (light yellow); %SRD value < 30%, acceptable (light green); >30%, poor (light yellow). Abbreviations: CI, confidence interval; FM, fibromyalgia; HD, Handgrip Test Dominant Side; HND, Handgrip Test Non-Dominant Side; ICC, intraclass correlation; LoA, Limits of Agreement; SD, standard deviation; SEM, standard error of measurement; SRD, smallest real difference; 6MWT, 6 min walking test; 8 FUGT, 8 feet up and go test.

**Table 5:** Reliability of PROMs in FM total sample.

	Test	Retest	ICC (95%CI)	SEM	SEM%	SRD	SRD%	LoA
	Mean (SD)	Mean (SD)						
FM total (n = 87)								
FIQ-R TS (0–100)	73.76 (12.91)	73.76 (13.69)	0.919 (0.876–0.947)	3.79	5.13	10.49	14.22	−14.36; 14.35
Function (0–30)	21.27 (4.60)	21.38 (4.89)	0.898 (0.844–0.933)	1.52	7.11	4.20	19.70	−5.80; 5.58
Overall impact (0–20)	14.97 (4.14)	14.45 (4.20)	0.761 (0.634–0.844)	2.04	13.86	5.65	38.41	−6.67; 7.70
Symptoms (0–50)	37.52 (6.40)	37.94 (6.41)	0.894 (0.837–0.931)	2.09	5.53	5.78	15.32	−8.20; 7.37
WPI (0–19)	14.02 (2.79)	14.00 (3.11)	0.880 (0.817–0.922)	1.02	7.29	2.83	20.22	−3.78; 3.83
SSS (0–12)	9.77 (1.87)	9.58 (1.78)	0.819 (0.723–0.882)	0.78	8.03	2.15	22.24	−2.61; 3.00
SF-36 (0–100)								
Physical component	23.02 (5.34)	23.01 (4.87)	0.881 (0.818–0.922)	1.76	7.65	4.88	21.21	−6.56; 6.58
Mental component	41.56 (6.69)	41.61 (7.09)	0.894 (0.838–0.931)	2.24	5.39	6.22	14.95	−8.46; 8.35
HADS TS (0–42)	24.31 (6.92)	24.36 (7.08)	0.943 (0.913–0.963)	1.67	6.87	4.63	19.04	−6.43; 6.37
Anxiety (0–21)	12.91 (4.01)	12.78 (4.02)	0.923 (0.881–0.949)	1.11	8.67	3.09	24.04	−4.11; 4.36
Depression (0–21)	11.40 (3.89)	11.58 (3.84)	0.934 (0.899–0.957)	0.99	8.64	2.75	23.95	−3.96; 3.61

Note: ICC value and reliability level < 0.50, poor (red); 0.50–0.75, moderate (orange); 0.75–0.90, good (yellow); >0.90 excellent (green); %SEM value < 10%, acceptable (light green); >10%, poor (light yellow); %SRD value < 30%, acceptable (light green); >30%, poor (light yellow). Abbreviations: CI, confidence interval; FIQ-R TS, Fibromyalgia Impact Questionnaire Revised Total Score; FM, fibromyalgia; HADS TS, Hospital Anxiety and Depression Total Score; ICC, intraclass correlation; LoA, Limits of Agreement; PROM, patient-reported outcome measures; SD, standard deviation; SEM, standard error of measurement; SF-36, 36-Item Short Form Survey; SRD, smallest real difference; SSS, Symptom Severity Score; WPI, Widespread Pain Index.

**Table 6:** Reliability of PROMs in FM patient subgroups.

	Test	Retest	ICC (95%CI)	SEM	SEM%	SRD	SRD%	LoA
	Mean (SD)	Mean (SD)						
Actively working (n = 30)								
FIQ-R TS (0–100)	65.81 (12.31)	64.73 (13.10)	0.909 (0.809–0.957)	3.83	5.87	10.62	16.28	−13.37; 15.53
Function (0–30)	18.60 (4.84)	18.58 (4.83)	0.885 (0.758–0.945)	1.64	8.82	4.54	24.45	−6.16; 6.21
Overall impact (0–20)	13.27 (3.95)	11.80 (3.70)	0.631 (0.225–0.824)	2.32	18.54	6.44	51.38	−6.32; 9.25
Symptoms (0–50)	33.94 (6.05)	34.35 (6.44)	0.875 (0.737–0.940)	2.21	6.47	6.12	17.92	−8.66; 7.84
WPI (0–19)	12.30 (2.95)	11.67 (2.73)	0.799 (0.578–0.904)	1.27	10.62	3.53	29.45	−3.93; 5.19
SSS (0–12)	8.73 (2.27)	8.33 (1.79)	0.805 (0.590–0.907)	0.90	10.51	2.48	29.13	−2.84; 3.64
SF-36 (0–100)								
Physical component	24.60 (6.27)	24.74 (5.04)	0.881 (0.750–0.943)	1.95	7.91	5.41	21.92	−7.52; 7.25
Mental component	44.50 (5.30)	44.20 (6.36)	0.763 (0.502–0.887)	2.84	6.40	7.87	17.74	−9.86; 10.47
HADS TS (0–42)	21.17 (6.02)	21.07 (5.79)	0.876 (0.739–0.941)	2.08	9.85	5.76	27.29	−7.71; 7.91
Anxiety (0–21)	11.33 (3.44)	10.67 (3.43)	0.885 (0.759–0.945)	1.16	10.59	3.23	29.35	−3.65; 4.98
Depression (0–21)	9.83 (3.60)	10.40 (3.47)	0.854 (0.692–0.930)	1.35	13.35	3.74	37.01	−5.52; 4.39
Claiming disability (n = 30)								
FIQ-R TS (0–100)	77.77 (11.27)	77.73 (13.25)	0.865 (0.716–0.936)	4.50	5.79	12.49	16.06	−16.85; 16.93
Function (0–30)	23.25 (3.01)	22.98 (4.59)	0.793 (0.565–0.901)	1.73	7.48	4.79	20.73	−6.10; 6.64
Overall impact (0–20)	15.70 (3.75)	15.57 (4.16)	0.679 (0.325–0.847)	2.24	14.33	6.21	39.73	−7.62; 7.88
Symptoms (0–50)	38.82 (6.82)	39.18 (6.67)	0.869 (0.724–0.937)	2.44	6.26	6.77	17.35	−9.49; 8.77
WPI (0–19)	14.23 (2.33)	14.80 (2.67)	0.867 (0.721–0.937)	0.91	6.28	2.53	17.41	−3.93; 2.80
SSS (0–12)	10.00 (1.60)	10.07 (1.26)	0.745 (0.465–0.879)	0.72	7.20	2.00	19.95	−2.64; 2.50
SF-36 (0–100)								
Physical component	21.89 (4.30)	22.23 (3.38)	0.835 (0.654–0.922)	1.56	7.07	4.32	19.60	−6.09; 5.41
Mental component	39.86 (7.21)	40.35 (7.63)	0.938 (0.870–0.971)	1.85	4.61	5.12	12.77	−7.56; 6.58
HADS TS (0–42)	26.50 (7.26)	26.13 (7.82)	0.963 (0.922–0.982)	1.45	5.51	4.02	15.28	−5.28; 6.02
Anxiety (0–21)	13.83 (4.25)	13.63 (4.20)	0.926 (0.844–0.965)	1.15	8.37	3.19	23.20	−4.21; 4.61
Depression (0–21)	12.67 (4.06)	12.50 (4.16)	0.967 (0.930–0.984)	0.75	5.93	2.07	16.44	−2.75; 3.08
Disability (n = 27)								
FIQ-R TS (0–100)	78.14 (11.37)	79.40 (9.36)	0.923 (0.831–0.965)	2.88	3.65	7.97	10.12	−12.17; 9.65
Function (0–30)	22.05 (4.53)	22.72 (3.96)	0.934 (0.856–0.970)	1.09	4.87	3.02	13.50	−4.81; 3.48
Overall impact (0–20)	16.04 (4.30)	16.15 (3.36)	0.855 (0.681–0.934)	1.46	9.06	4.04	25.12	−5.58; 5.36
Symptoms (0–50)	40.06 (4.41)	40.54 (4.06)	0.872 (0.719–0.942)	1.52	3.76	4.20	10.42	−6.10; 5.14
WPI (0–19)	15.70 (1.90)	15.70 (2.37)	0.862 (0.697–0.937)	0.79	5.05	2.20	14.00	−2.98; 2.98
SSS (0–12)	10.67 (0.92)	10.41 (1.53)	0.646 (0.222–0.838)	0.73	6.92	2.02	19.17	−2.27; 2.79
SF-36 (0–100)								
Physical component	22.52 (5.02)	21.97 (5.66)	0.892 (0.763–0.951)	1.75	7.89	4.86	21.87	−6.02; 7.12
Mental component	40.18 (6.59)	40.14 (6.66)	0.904 (0.789–0.956)	2.05	5.11	5.69	14.17	−7.79; 7.86
HADS TS (0–42)	25.37 (6.40)	26.04 (6.41)	0.952 (0.895–0.978)	1.40	5.46	3.89	15.13	−6.02; 4.69
Anxiety (0–21)	13.63 (3.91)	14.19 (3.57)	0.932 (0.851–0.969)	0.98	7.01	2.70	19.43	−4.26; 3.15
Depression (0–21)	11.74 (3.53)	11.85 (3.65)	0.952 (0.896–0.978)	0.79	6.67	2.18	18.48	−3.15; 2.93

Note: ICC value and reliability level < 0.50, poor (red); 0.50–0.75, moderate (orange); 0.75–0.90, good (yellow); >0.90 excellent (green); %SEM value < 10%, acceptable (light green); >10%, poor (light yellow); %SRD value < 30%, acceptable (light green); >30%, poor (light yellow). Abbreviations: CI, confidence interval; FIQ-R TS, Fibromyalgia Impact Questionnaire Revised Total Score; FM, fibromyalgia; HADS TS, Hospital Anxiety and Depression Total Score; ICC, intraclass correlation; LoA, Limits of Agreement; PROM, patient-reported outcome measures; SD, standard deviation; SEM, standard error of measurement; SF-36, 36-Item Short Form Survey; SRD, smallest real difference; SSS, Symptom Severity Score; WPI, Widespread Pain Index.

## Data Availability

The original contributions presented in this study are included in the article. Further inquiries can be directed to the corresponding author.

## Abbreviations

The following abbreviations are used in this manuscript:

FM	Fibromyalgia
ACR	American College of Rheumatology
PBTs	Performance-based tests
PROMs	Patient-reported outcome measures
6MWT	6 min walk test
COSMIN	Consensus-based Standards for the selection of health Measurement Instruments
BMI	Body mass index
WPI	Widespread Pain Index
SSS	Symptom Severity Scale
FIQR	Revised Fibromyalgia Impact Questionnaire
SF-36	36-item Short-Form Health Survey
HADS	Hospital Anxiety and Depression Scale
ICF	International Classification of Functioning, Disability and Health
ICC	Intraclass correlation coefficient
SEM	Standard error of measurement
SRD	Smallest real difference
LoA	Limits of Agreement
EWGSOP2	The European Working Group on Sarcopenia in Older People 2
